# Sclerotic lumbar chordoma: A case report

**DOI:** 10.1016/j.radcr.2022.05.055

**Published:** 2022-06-29

**Authors:** Owaiz Ansari, Rohit Anand, Kevin Christopher Serdynski, Serra Aktan, Brett Ploussard, Emad Allam

**Affiliations:** a Department of Radiology and Medical Imaging, Loyola University Medical Center, 2160 S 1st Ave, Maywood, IL 60153, USA; b Rowan University School of Osteopathic Medicine, 42 E Laurel Rd, Stratford, NJ 08084, USA

**Keywords:** Chordoma, Lumbar spine, Ivory vertebra, FDG, fluorodeoxyglucose, SUV, standardized uptake value, Tc99m MDP, technetium 99m methylene diphosphonate, MMP-1, matrix metalloproteinase-1, uPA, urokinase plasminogen activator

## Abstract

Chordoma is a rare tumor, often occurring in the cervical spine and sacrococcygeal spine with a lytic appearance, but rarely in the thoracolumbar spine. Chordomas can occasionally be sclerotic and are included in the differential diagnosis for an ivory vertebra. We present a case of a sclerotic chordoma in an upper lumbar vertebral body with corresponding multimodality imaging. This case demonstrates that chordoma should be a concern for an older adult with a sclerotic vertebral lesion, particularly if it is a solitary lesion. Knowledge of the variable location and appearance of chordomas is critical so it is not mistaken for a metastasis.

## Background

Chordoma is a rare, slow-growing, malignant tumor originating from notochordal remnants. It most often occurs at the base of the skull, cervical spine, and sacrococcygeal spine, rarely occurring in the thoracolumbar spine. It typically has a destructive, lytic appearance on imaging. It occurs in adults in the fifth to seventh decades of life with an overall male predominance. Chordomas are often asymptomatic and only present clinically when the lesion is large and compressing local structures. The first line treatment is en bloc resection with wide surgical margins with or without adjuvant radiotherapy and chemotherapy. Radiation is typically used for patients that decline surgery, cannot achieve tumor-free surgical margins, or have metastatic disease [1]. However, the value of postoperative radiotherapy is unclear because chordomas are relatively radio-resistant [2]. Chordomas have high rates of local recurrence and distant metastases may occur. Thus, it is critical to establish a correct diagnosis early. Imaging can help guide diagnosis and management. The average life expectancy for patients with chordoma is 6 years, with a 5-year survival rate of 70% and 10-year survival rate of 40%.

Here, we present an unusual case of a chordoma of the L2 vertebral body with a sclerotic appearance, occurring in a 57-year-old female. Such a lesion may be mistaken for a metastasis, and awareness of the variable location and appearance of chordomas is important so that it can be included in the differential diagnosis.

## Case presentation

A 57-year-old female presented with intermittent lower back pain, exaggerated with increased activity. Occasionally, there was radiating pain to the left buttock and numbness and tingling in the bilateral thighs. She reported no weakness or bowel incontinence. She reported no significant weight loss, fevers, or chills. She had no significant past medical, surgical, or family history.

**Fig. 1:**
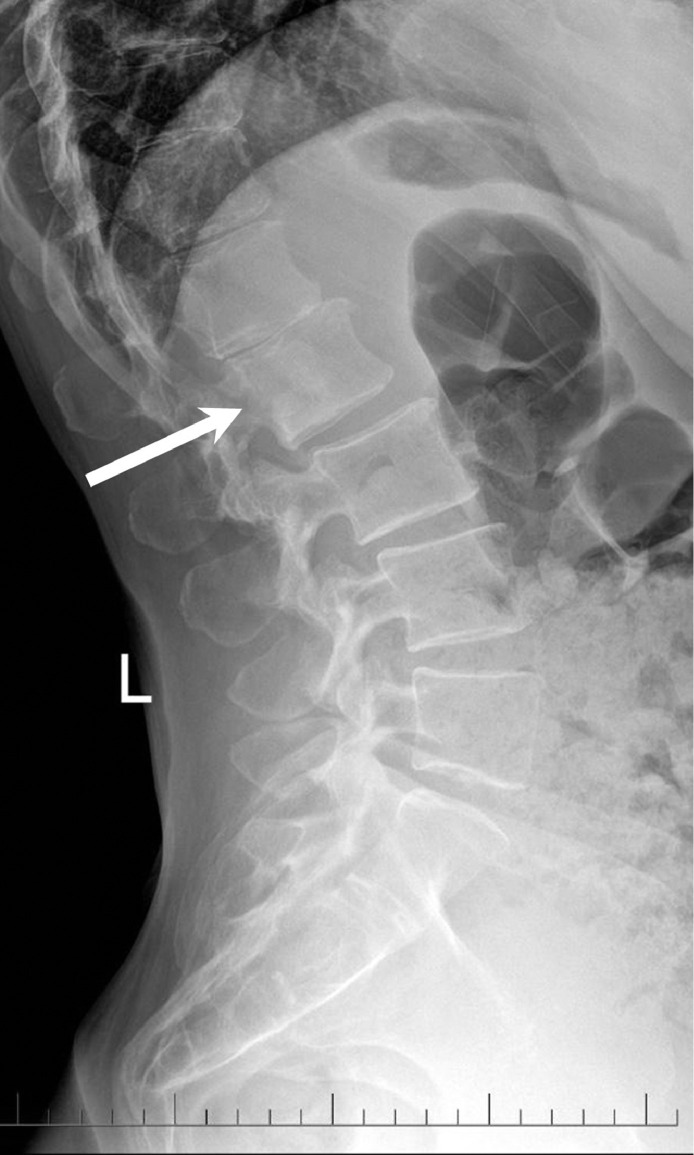
Lateral radiograph of the lumbar spine demonstrates subtle sclerosis in the posterior half of the L2 vertebral body (arrow). Intervertebral disc space narrowing is noted at T11-T12 and L1-L2. There is grade 1 retrolisthesis at L1-L2, L2-L3, and L3-L4.

**Fig. 2:**
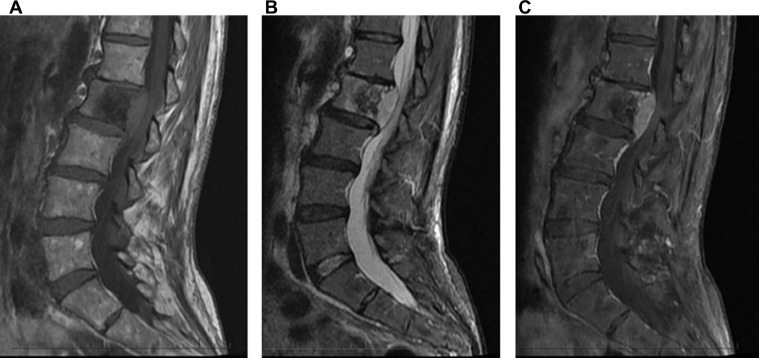
MRI of the lumbar spine including (A) T1, (B) STIR, and (C) postcontrast T1 fat-saturated sagittal images demonstrate a T1 hypointense and STIR heterogeneous lesion predominantly in the posterior aspect of the L1 vertebral body, extending from superior to inferior endplate and with irregularity of the posterior cortex. The anterior aspect of the vertebral body is spared. There is an associated epidural soft tissue component along the posterior aspect of the L2 vertebral body which is T1 isointense (relative to disk) and STIR hyperintense with homogenous enhancement; this results in spinal canal stenosis with compression of the thecal sac and conus medullaris/cauda equina at this level. There is heterogeneous marrow signal in the other vertebrae without a focal lesion. Degenerative disc disease is noted at L1-L2. The conus medullaris terminated at the mid-L2 level in this patient.

**Fig. 3:**
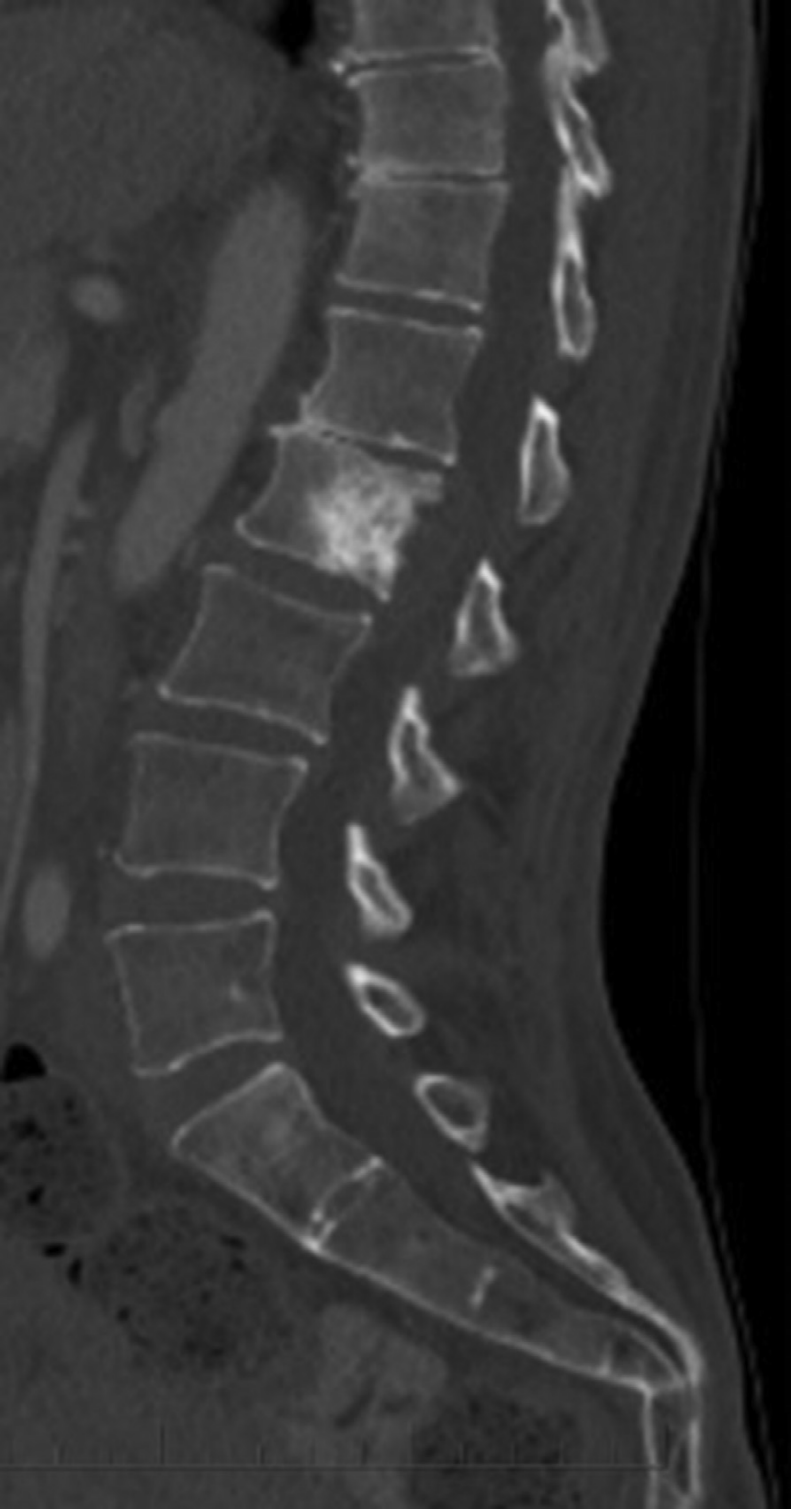
Mid sagittal CT image demonstrates a sclerotic lesion in the L2 vertebral body with irregular margins and destruction of the posterior cortex. No mineralization is seen in the epidural soft tissue component. No other suspicious lesion was identified on this CT of the chest, abdomen, and pelvis with IV contrast.

**Fig. 4:**
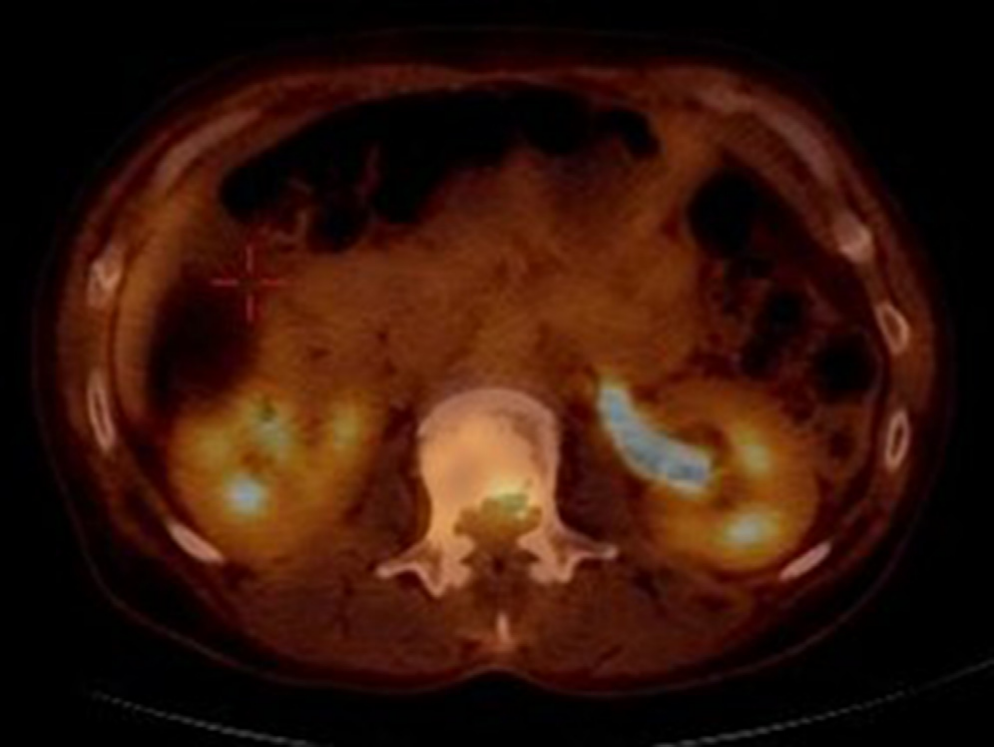
PET/CT demonstrates increased FDG uptake in the posterior aspect of the L2 vertebral body and associated soft tissue mass with SUV max of 4.2. Physiologic radiotracer uptake is seen in the bilateral kidneys and left ureter. No other hypermetabolic lesion was identified on this PET/CT.

**Fig. 5:**
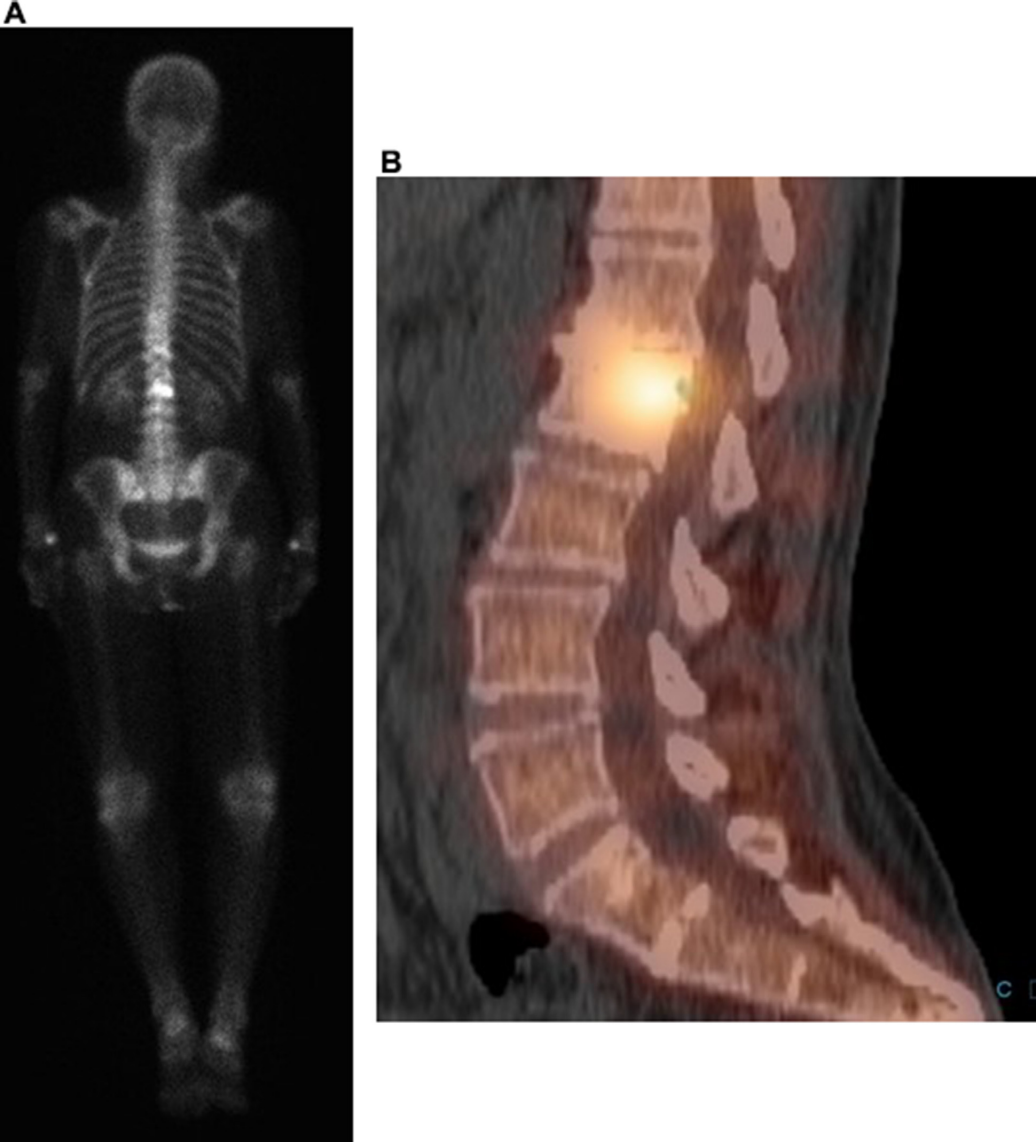
(A) Tc99m MDP whole body bone scan demonstrates focal increased radiotracer uptake in the L2 vertebral body, corresponding to the known lesion. (B) This is confirmed on concurrent SPECT-CT. No additional suspicious osseous uptake is seen on this bone scan.

The patient underwent physical therapy without significant improvement. Radiographs of the lumbar spine demonstrated sclerosis in the posterior aspect of the L2 vertebral body and degenerative changes (Fig. 1). Subsequent MRI revealed a sclerotic lesion in the L2 vertebral body with epidural extension and spinal canal stenosis (Fig. 2). CT of the chest, abdomen, and pelvis, PET/CT, and bone scan revealed no other lesion (Fig. 3-5). Screening mammograms with tomosynthesis demonstrated heterogeneously dense breasts with no evidence of malignancy. Differential diagnosis based on imaging included metastasis, lymphoma, chordoma, and aggressive hemangioma.

**Fig. 6:**
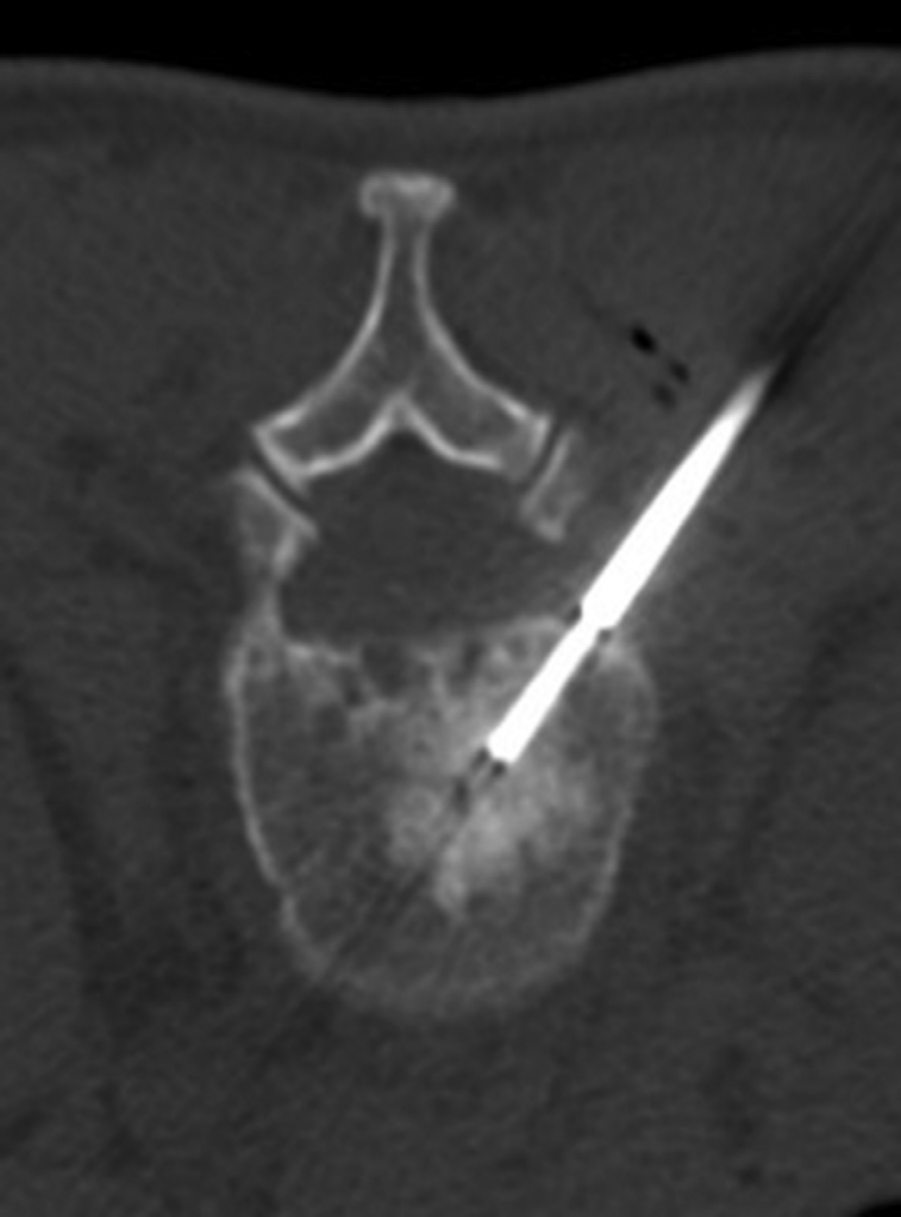
Representative image showing CT-guided biopsy of the L2 vertebral body sclerotic lesion from a right posterolateral approach.

CT-guided biopsy of the sclerotic osseous lesion at L2 was consistent with a chordoma (Fig. 6). The patient subsequently underwent 2-stage surgery including L2 spondylectomy with anterior reconstruction and T11-L5 posterior spinal fusion. The patient was on steroids for 6 weeks preoperatively. No radiation therapy or chemotherapy was administered.

## Discussion

Chordoma is a rare neoplasm originating from the primitive notochord. Their incidence is low, about 0.08 per 100,000 people per year. Lumbar spine chordomas account for only 6% of all chordomas [3]. They are seen more often in males than in females, at a 2:1 ratio overall [4]. Although most are slow-growing, they are locally invasive. If they metastasize, this usually occurs to the lungs, liver, skin, distal bone, and soft tissue [1,4]. Unfortunately, rates of local recurrence are high, but are decreased with successful en-bloc resection. Adjuvant radiotherapy does not prevent recurrence but is known to increase the disease-free period [5]. Chemotherapy of chordomas can also be considered and often consists of anthracycline, cisplatin, alkylating agents and camptothecin analogues [6].

The typical imaging findings of vertebral chordoma are well-described in the literature. On CT, chordomas typically appear in the axial skeleton as a lytic lesion. Lesions are usually well-circumscribed due to slow growth and may include calcification and marginal sclerosis (Hatem, Maclean). On T1-weighted MRI, chordomas appear as a low to intermediate-intensity lesion that may extend into or encase the adjacent neurovascular structures [7,8]. On T2-weighted MRI, chordomas appear as a high-intensity lesion, although the exact reason is unclear. The most commonly cited cause is intratumoral fluid or the presence of proteinaceous, mucinous material within the lesion [7,8].

Chordomas can occasionally be sclerotic and are included in the differential diagnosis for an ivory vertebra. This case demonstrates that chordoma should be considered in the differential diagnosis for an older adult with a sclerotic vertebral lesion, particularly if it is a solitary lesion.

On pathology, chordomas macroscopically appear lobulated and can grow up to 15 cm. They often contain calcification and hemorrhage and can erode through bony structures. Pathognomonic physaliphorous cells are seen in chordomas [8]. Diagnosis of conventional chordoma can be aided with certain markers such as cytokeratin and S-100. Galectin-3 expressed in the primitive notochord can be used to distinguish a chordoma from a chondrosarcoma. Additionally, expression of the brachyury gene has become an important marker for diagnosis and should be tested if chordoma is suspected [4]. The brachyury stain in our patient was negative, likely due to decalcification during processing of the specimen, which made the diagnosis of chordoma more challenging. Rare occurrences of familial chordoma have shown a common gene duplication of the brachyury transcriptional regulator [6]. Poor prognosis has been tied to expression of MMP-1 and uPA which are also correlated to increased local invasion [4].

## Conclusion

Despite increasing prevalence of spinal metastatic disease, primary neoplasms of the spine do occur and should be recognized. Chordomas are rare primary neoplasms of the spine that arise from embryological remnants of the notochord. Our case serves as an illustrative example of why chordoma should be included in the differential diagnosis of a vertebral lesion despite atypical location and imaging findings.
